# A Real-World Study in Non-Functional Adrenal Tumours: Refining Central DXA Results

**DOI:** 10.3390/jcm15114114

**Published:** 2026-05-26

**Authors:** Nina Ionovici, Alexandra-Ioana Trandafir, Oana-Claudia Sima, Mihai Costachescu, Mara Carsote

**Affiliations:** 1 Occupational Medicine Department, University of Medicine and Pharmacy of Craiova, 200349 Craiova, Romania; nina.ionovici@umfcv.ro (N.I.);; 2 PhD Doctoral School, “Carol Davila” University of Medicine and Pharmacy, 020021 Bucharest, Romania; 3 Department of Clinical Endocrinology V, “C.I. Parhon” National Institute of Endocrinology, 011863 Bucharest, Romania; 4 Department of Radiology and Medical Imaging, “Dr. Carol Davila” Central Military University Emergency Hospital, 010825 Bucharest, Romania; 5 Department of Endocrinology, “Carol Davila” University of Medicine and Pharmacy, 020021 Bucharest, Romania

**Keywords:** chronic disease, marker, hormone, lab testing, bone mineral density, DXA, tumour, cortisol, menopause, T-score, ACTH, osteocalcin

## Abstract

**Background**: Osteoporosis, a chronic disease with a major epidemiologic impact amid menopause might be aggravated by co-ailments such as adrenal tumours, with an increasing incidence due to a larger access to imaging evaluation. The objective was to evaluate bone profile in relationship with adrenal profile in non-functioning adrenal tumours (NFATs), based on menopausal DXA categories (osteoporosis, osteopenia and normal). **Methods**: A retrospective real-life study was conducted amid a cross-sectional analysis in anti-osteoporotic drugs naïve subjects. Adrenal profile included baseline morning plasma cortisol (base-cortisol), second-day cortisol (DST-cortisol) after 1 mg dexamethasone testing, ACTH, and largest tumour diameter at CT (D-CT). **Results**: Ninety-five patients (mean age 61.59 ± 7.83 years) had 24.21% osteoporosis, 47.37% osteopenia, and 28.42%—normal DXA. Base-cortisol, DST-cortisol, ACTH and D-CT were similar between the groups. Total serum calcium was lower in osteoporosis versus osteopenia, versus normal DXA (9.26 ± 0.52 versus 9.61 ± 0.41 mg/dL, *p* = 0.005, respectively, 9.79 ± 0.47 mg/dL, *p* < 0.001). Osteocalcin, respectively, CrossLaps were elevated in osteoporosis versus osteopenia. MACS prevalence was 27.37% (no between-group difference). Osteoporosis group: CrossLaps correlated with DST-cortisol (r = −0.550, *p* = 0.019). Multiple linear regression model to predict lumbar BMD explained 47.1% of the variance in lumbar BMD (R^2^ = 0.471). ACTH was an independent variable for lumbar BMD (*p* = 0.007). BMI represented the main influential contributor to this model having the highest β of 0.490, and it also explained 49.1% (R^2^ = 0.491) of total hip BMD variation. **Conclusions**: This study emphasises a heterogeneous connection between adrenal profile in NFATs and clinical evaluation of the bone status. More comprehensive prospective studies are mandatory to assess this multifactorial bone–adrenal interplay in order to improve the overall management.

## 1. Introduction

Increased life span as a distinct feature of the aging process in modern society includes an increasing menopausal population, thus, the importance of understanding endocrine ailments that potentially interfere with the menopausal status, including osteoporosis and (osteoporotic) fragility fractures [1,2]. The burden of menopause-related (primary) osteoporosis is elevated by the co-presence of multiple contributors and risk factors, other than the traditional panel (e.g., smoking, corticotherapy, type 1 diabetes mellitus, etc.), such as the presence of mild cortisol production by an adrenal tumour (mostly detected across an incidental imaging scan), which does not represent the typical/traditional Cushing’s syndrome [3,4,5,6].

Currently, adrenal incidentalomas have an increasing detection rate due to easy access to various imaging assessments for thoracic, abdominal and pelvic conditions. The peak incidence is age-dependent; starting at the age of 40, women are more affected than males [7,8,9,10]. Approximately 60% to 90% (depending on the study population) of these incidentalomas are non-functioning from a hormonal perspective. However, 20% up to 40% of the non-functioning adrenal tumours (NFATs) actually display small hormonal activity, namely, mild autonomous cortisol secretion (MACS) [11,12,13].

MACS-positive neoplasia and to a lesser extent MACS-negative NFATs have been found to be associated with a higher cardiovascular and metabolic risk and even a higher mortality rate when compared to the general age- and menopause-matched population. On the other hand, some studies pinpointed that an elevated risk of bone loss is present, particularly in certain population subgroups, such as menopausal women [12,13].

To date, heterogeneous results have been found with respect to the impairment of bone turnover markers, mineral metabolism assays, and bone mineral density (designated as BMD), as provided according to a GE Lunar Prodigy (as we applied in this study) central Dual-Energy X-Ray Absorptiometry (DXA) scan, while the direct correlation with the adrenal hormonal panel [cortisol overproduction or adrenocorticotropic hormone (ACTH) suppression] and the adrenal tumour size is still an open matter. Current data are less common than the studies involving cardio-metabolic traits in these tumour masses; so, the overall level of statistical evidence remains low. Overall, some authors confirmed a higher risk of osteoporosis and fractures in individuals with MACS, but others did not. Other data suggested that patients with adrenal tumours who are classified as MACS-negative might show certain osseous and non-osseous risk, because the current definition criteria are still lacking multimodal parameters, other than just using the cutoff for cortisol [14,15].

### Objective

The working hypothesis is that adrenal hormonal profile might be distinctly displayed in patients with the three main DXA categories (osteoporosis, osteopenia and normal), and these adrenal variables might show a distinct correlation with the mineral metabolism assays/bone turnover markers and bone mass if one individual is registered in a distinct DXA group.

Our endpoint is to perform an analysis of bone profile (mineral metabolism assays, bone turnover markers, and BMD) in relationship with the adrenal profile (cortisol and ACTH assays, respectively, adrenal imaging findings) in menopausal women diagnosed with NFATs, based on DXA categories (osteoporosis, osteopenia and normal).

The novelty of this real-world analysis comes from orienting the analysis of adrenal tumours on the standard WHO-DXA categories, rather than cortisol-oriented MACS/non-MACS profile in a landscape of the paucity of published data in the bone–adrenal crosstalk.

## 2. Methods

We conducted a cross-sectional, retrospective, and real-life analysis, in a single department of a tertiary centre of endocrinology (university hospital). The Ethical Committee of the hospital and the university approved the study (as shown below).

The study population included consecutive menopausal women (inpatients) who were confirmed to have unilateral or bilateral NFATs and had at least one hospitalisation between January 2023 and August 2025. Inclusion criteria at the moment of NFAT diagnosis were patient age ≥ 50; menopause (at least 12 months since the last menses), written informed consent during hospitalisation (according to the hospital rules for anonymous use of the medical records), and the diagnosis of NFAT based on the specific endocrine assessment (hormonal assays and imaging evaluation).

The exclusion criteria were as follows: drugs against osteoporosis (across life span) or fracture risk reduction (e.g., bisphosphonates, denosumab, teriparatide, romosozumab, calcitonin); prior or current diagnosis of malignancies (including adrenal cancer), bone metabolic disorders, type 1 diabetes, functioning (secretor) endocrine tumours (e.g., somatotropinoma, corticotropinoma, prolactinoma, parathyroid tumour, pheochromocytoma, aldosteronoma), neuroendocrine neoplasia, chronic kidney disease, endogenous or exogenous Cushing’s syndrome, uncontrolled thyrotoxicosis or hypothyroidism; unilateral or bilateral adrenalectomy; clinically suspected Cushing’s syndrome; hormone replacement therapy (oestrogens with/without progesterone), corticotherapy or insulin therapy (current or previous one year); suspected or confirmed non-benign imaging features at computed tomography (irregular shape, vessel invasion, local metastases); adrenal cysts. Individuals who had incomplete data at the central DXA evaluation and computed tomography were also ruled out.

The subjects underwent a biochemical and hormonal (particularly, adrenal) evaluation. The collected demographic parameters were age (years), menopause duration (years since last menstruation), (current or previous) diagnosis of type 2 diabetes, dyslipidaemia (of any type), and hypertension. The body mass index (BMI = height and weight) was calculated in kg/sqm. The biochemistry profile also included the following: fasting glycaemia (via photometry method, provided by an Abbott kit (Abbott, Abbott Park, IL, USA); normal: 80–115 mg/dL) and glycated haemoglobin A1C [turbidimetry method (Roche manufacturer, Basel, Switzerland); normal: 4.8–5.9%] at the moment of NFAT diagnosis and DXA evaluation.

The adrenal profile included the hormonal panel, specifically, ACTH, and cortisol from plasma (in the morning), as well as the plasma morning cortisol after a 1 mg dexamethasone test (DST), which was administered at 11 p.m. Cortisol and ACTH were tested according to ECLIA/Roche detection (normal morning baseline unsuppressed levels of 7.2–63.3 pg/mL, respectively, 4.82–19.5 µg/dL).

The patients with a second-day plasma cortisol of ≥5 µg/dL were excluded. MACS-positive was defined when this cortisol assay showed a value >1.8 µg/dL and <5 µg/dL [14]. The adrenal imaging profile included the computed tomography-based data concerning unilateral or bilateral NFAT/NFATs and the largest tumour diameter of the single tumour or the largest diameter between both tumours in the case of bilateral NFATs.

The bone profile included blood assays of the mineral metabolism (total serum calcium, phosphorus, magnesium—all based on the photometry (Roche for calcium or Abbott for phosphorus and magnesium) method, normal ranges: 8.8–10.2 mg/dL, 2.3–4.7 mg/dL, respectively, 1.6–2.6 mg/dL), as well as hormones, e.g., parathormone (PTH, based on ECLIA (Roche) method of detection; normal: 17.3–74.1 pg/mL) and vitamin D (specifically, 25-hydroxyvitamin D, based on CLIA (DiaSorin, Saluggia, Italy) assessment; normal > 30 ng/mL). Blood markers included osteocalcin and P1NP (bone formation; ECLIA (Roche) method, normal: 15–46 ng/mL, respectively, 20.25–76.31 ng/mL), and CrossLaps (bone resorption; ECLIA (Roche) method, normal: 0.33–0.783 ng/mL). In addition, total alkaline phosphatase was tested as a bone formation marker, as well, by applying the photometry method of detection with a Roche kit/manufacturer.

DXA was done at the L1–L4 lumbar spine, as well as the hip (femoral neck and total hip), providing the BMD and associated T-score. The study groups were designated as A, B or C, if the patients were confirmed to have osteoporosis (group A), osteopenia (group B) or a normal T-score at central DXA (group C), based on the WHO categories [1,2]. The lumbar spine DXA provided the trabecular bone score (TBSiNsight software, ver. 3). The prevalence of fragility (osteoporotic/low trauma or spontaneous) fractures was explored based on searching in the medical records; in addition, each individual underwent a screening profile X-ray at the level of the thoracic–lumbar spine during hospitalisation.

For statistical analysis, we used SPSS v.29.0.2.0 (IBM), Excel v.16.102.2 (Microsoft), and GraphPad Prism v.10.6.0. We introduced the following: continuous variables [average ± standard deviation (SD) or median and quartiles (Q1, Q3)], categorical variables (number/percentage), as well as comparisons (*t*-test or Mann–Whitney U test) between two independent groups, and across three groups (A, B and C) with one-way ANOVA or Kruskal–Wallis test, followed by post hoc pairwise comparisons. Chi-square or Fisher’s exact test allowed the comparison of categorical variables.

Correlations (Kendall’s tau coefficient) were analysed between the adrenal parameters, bone turnover markers, and BMD within each group. *p* < 0.05 showed statistical significance. Adjustments for multiple comparisons (Bonferroni) correction have been applied.

Multiple linear regressions were used to assess the association between the dependent and independent variables. For each independent variable, unstandardised (B ± SE) and standardised coefficients (β) indicated the expected change in the dependent variable. The R^2^ coefficient quantified the proportion of variance explained by the model.

## 3. Results

**Figure 1:**
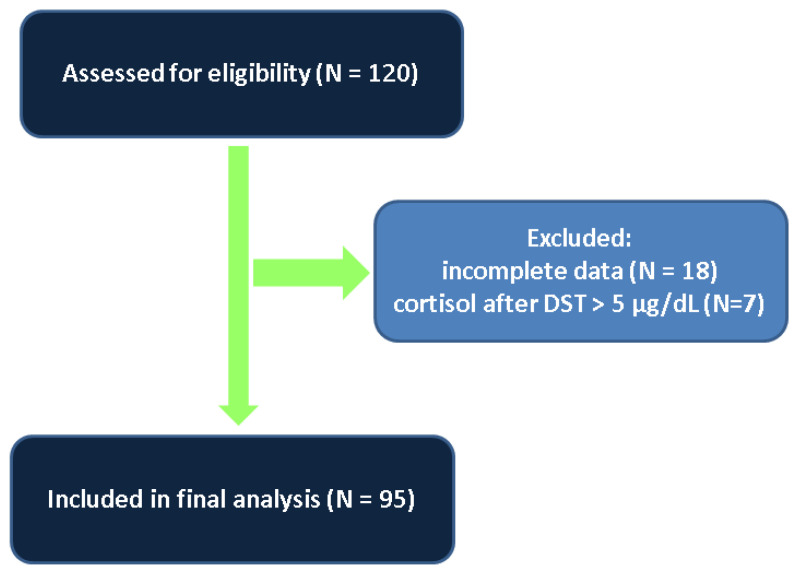
Study diagram.

There were 95 participants with a mean age of 61.59 ± 7.83 years and average menopause duration of 13.88 ± 8.24 years. In total, 24.21% patients were diagnosed with osteoporosis (group A), 47.37% were diagnosed with osteopenia (group B), and 28.42% had a normal BMD/T-score (group C) (Figure 1).

The age and menopausal time period were similar among these three groups.

The BMI was statistically significant higher in group C (normal DXA) versus B (osteopenia) (33.24 ± 7.52 versus 29.60 ± 4.89 kg/sqm, *p* = 0.003).

The glycated haemoglobin A1c was increased in group C (normal DXA) versus group B (osteopenia) (6.72 ± 1.82% versus 5.86 ± 0.55%, *p* = 0.05).

The median (Q1, Q3) value of the morning ACTH was 11.88 (7.86, 16.28) pg/mL, respectively, the mean baseline morning plasma cortisol was 14.19 ± 6.53 µg/dL and the median plasma cortisol after 1 mg DST was 1.82 (1.13, 3.02) µg/dL. The average largest tumour diameter was 2.32 ± 0.98 cm. These adrenal (hormonal and imaging) parameters were similar in group A, B and C.

The total serum calcium was significantly reduced in group A (osteoporosis) versus B (osteopenia) (9.26 ± 0.52 versus 9.61 ± 0.41 mg/dL, *p* = 0.005) and versus group C (normal DXA) (9.79 ± 0.47 mg/dL, *p* < 0.001).

Osteocalcin (mean of 31.95 ng/mL) and CrossLaps (average of 0.65 ng/mL) were higher in group A than group B at 20.30 ± 8.71 ng/mL (*p* = 0.017), respectively, 0.41 ± 0.19 ng/mL, and group C at 22.71 ± 10.56 ng/mL, respectively, at 0.47 ± 0.24 ng/mL (*p* < 0.001, *p* = 0.042, respectively, *p* = 0.036) (Table 1).

**Figure 2:**
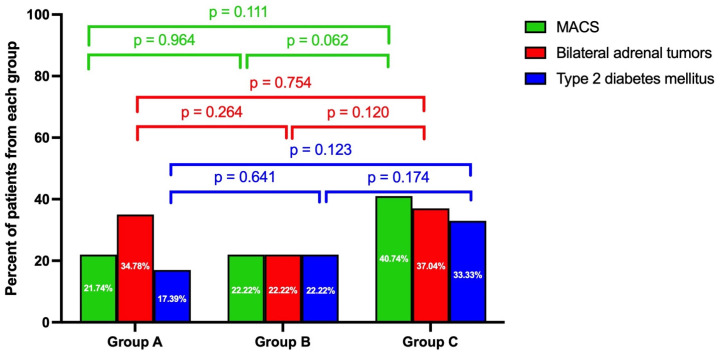
Prevalence of MACS-positive profile, bilateral adrenal tumours and type 2 diabetes within group A, group B and group C.

The global MACS prevalence was found to be 27.37%, respectively, at 24.21% for type 2 diabetes (no between-group difference) (Figure 2).

**Figure 3:**
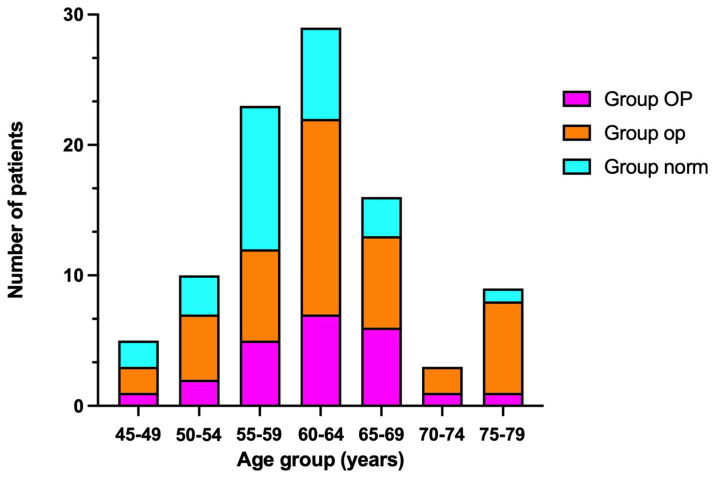
Stacked bar chart for the number of subjects from groups A, B and C within age group.

The ages within the studied groups (A, B and C) were similar (Table 2, Figure 3).

In the group with osteoporosis, ACTH showed statistically significant negative correlations between the baseline value and CrossLaps (r = −0.398, *p* = 0.048).

In the group with osteoporosis, ACTH was positively associated with 25-hydroxyvitamin D levels (r = 0.448, *p* = 0.02), as well as negatively with parathormone (r = −0.564, *p* = 0.016).

On the other hand, in the same group, the value of CrossLaps was negatively correlated with the level of cortisol following DST (r = −0.550, *p* = 0.019) (Table 3).

In the group with osteopenia, ACTH was found to be negatively correlated with P1NP (r = −0.367, *p* = 0.048) and with serum phosphorus (r = −0.311, *p* = 0.012) (Table 4).

In the group with normal results at DXA, the morning plasma cortisol showed a significant and negative correlation with osteocalcin (r = −0.391, *p* = 0.019).

In patients with normal BMD, this cortisol associated with the femoral T-score (r = −0.485, *p* = 0.006).

The level of cortisol after DST displayed two negative correlations: with serum phosphorus (r = −0.528, *p* = 0.01) and with parathormone (r = −0.515, *p* = 0.02).

The largest tumour diameter correlated with osteocalcin (r = −0.427, *p* = 0.011), with P1NP (r = −0.427, *p* = 0.017) and with serum phosphorus (r = −0.467, *p* = 0.007) (Table 5).

The multiple linear regression model used to predict the value of the lumbar BMD explained 47.1% of the variance in the lumbar BMD (R^2^ = 0.471).

The lumbar BMD in the absence of predictors was 1.067 ± 0.347 g/cm^2^ (*p* = 0.007). The BMI showed a statistically significant influence on the level of the lumbar BMD, showing that an increase in BMI of 1 kg/sqm leads to an elevation in lumbar BMD of 0.014 ± 0.006 g/cm^2^ (*p* = 0.030). The baseline ACTH was also a statistically significant independent variable for lumbar BMD, with an increase in baseline ACTH of 1 pg/mL leading to a decrease in lumbar BMD of −0.015 ± 0.005 g/cm^2^ (*p* = 0.007).

BMI remained the most influential contributor in the model with the highest β of 0.490 (Table 6).

The multiple linear regression model was applied in order to predict other sites at DXA; specifically, the level of the femoral neck BMD explained 58.3% of the variation in femoral neck BMD (R^2^ = 0.583).

The femoral neck BMD (when all independent variables were 0) was 1.322 ± 0.334 g/cm^2^ (*p* = 0.002).

The BMI was not a contributor to the femoral neck BMD: an increase in BMI of 1 kg/sqm led to an increase in femoral neck BMD of 0.011 ± 0.005 g/cm^2^ (*p* = 0.062).

In addition, the value of age, years since menopause, baseline ACTH, second-day plasma cortisol after 1 mg DST and the largest tumour diameter were not correlated with the femoral neck BMD in this model (Table 7).

The multivariate regression for the total hip BMD prediction explained 49.1% of the variation in the total hip BMD (R^2^ = 0.491). When all other contributors were 0, the total hip BMD was 0.853 ± 0.402 g/cm^2^, with borderline significance (*p* = 0.052).

An increase in BMI of 1 kg/sqm led to a statistically significant increase in the total hip BMD of 0.020 ± 0.007 g/cm^2^. The others independent variables were not found statistically significant as contributors to the total hip BMD in this model (Table 8).

## 4. Discussion

The endpoint of this real-world study was to examine consecutive menopausal females co-diagnosed with NFATs and osteoporosis/osteopenia (versus normal DXA) regarding potential correlations between the bone and the adrenal profile. To date, only a limited number of studies concerning this distinct area in the NFATs domain have been published, as opposed to numerous data regarding the evaluation of the cardiovascular risk in NFTAs [14,16,17,18].

Overall, we identified that 71.58% of the patients had non-normal DXA at a mean age of 61.59 ± 7.83 years, a rate increase compared to general population but with a relatively low fracture prevalence (6%) [14,19]. Of note, we only included subjects who were not under any medication to prevent osteoporosis, although a small subgroup showed a history of (spontaneous/low-trauma) fragility fractures during menopause.

One fifth of the subjects suffered from type 2 diabetes mellitus without between-group rate differences, but a higher glycated haemoglobin A1c was identified in subjects with normal DXA versus those with osteopenia (6.72 ± 1.82% versus 5.86 ± 0.55%, *p* = 0.05). In addition, a prevalence of 77.89%, respectively, 78.95%, for the diagnosis of arterial hypertension, respectively, dyslipidaemia, confirmed prior studies showing an increased rate of cardio-metabolic ailments in adults with NFATs [20,21]. The mean BMI was suggestive for obesity (30.05 ± 6.01 kg/sqm, N = 95), and women with a normal T-score showed a statistically significant higher BMI when compared to osteoporotic females (33.24 ± 7.52 versus 26.90 ± 4.51 kg/sqm).

Moreover, the multiple linear regression models showed that BMI was the most important predictor of lumbar BMD, while, among specific adrenal features, ACTH was the single parameter to predict lumbar BMD to some extent. Rebelo et al. [21], for instance, showed that NFATs/MACS were single independent predictors for resistant hypertension or metabolic syndrome [21], but the general results concerning bone status prediction are less clear. From a broader perspective, Nakao et al. [22] pointed out that the steroid profile might correlate with bone health, but other metabolites potentially provide a more accurate prediction (e.g., 11-deoxycorticosterone), molecules that are not established as biomarkers in daily practice for NFATs diagnosis and surveillance [22]. Of note, liquid chromatography and mass spectrometry of the urinary steroids are not routinely used in every medical centre, but a more refined hormonal analysis might pinpoint complex interferences with the bone status [23]. In addition, noting the mean BMI values in the study population, it would be interesting to analyse whether the BMI influence on BMD might be similar in non-obese groups.

A MACS-positive profile (as shown by the level of the morning plasma cortisol assay after dexamethasone administration during the overnight screening test) is expected to provide the most important influence on the DXA-based BMD/T-score according to some studies [24,25]. The term of “MACS-related osteoporosis” was recently proposed; however, the bone influence is multifactorial in individuals harbouring this type of adrenal tumours [24]. Yet, we mention a recent meta-analysis (2024), which confirmed that patients with MACS-positive tumours had a 1.5- to 2-fold increase of osteoporosis, osteopenia or osteoporotic fracture risk when compared to those with MACS-negative tumours [25]. In this instance, the absence of differences in MACS might create bias due to the sample size or inadequate definition criteria from the osseous perspective.

As found in this analysis, the menopausal status, the age, and the presence of type 2 diabetes potentially influence DXA-BMD and/or bone turnover markers in addition to direct bone actions of higher cortisol [26,27,28]. Thus, a single cortisol assay might not capture the entire essence of the bone impairment. Other authors suggested that a basal measurement of dehydroepiandrosterone sulphate (DHEAS), which is expected to be lower in MACS-positive than MACS-negative tumours, could be applied as a screening test for a mild cortisol secretion and further estimations of the clinical consequences, including osseous [29]. In our study, the MACS rate (previously named “subclinical Cushing’s syndrome”) was similar in each DXA category. Generally, the current study confirmed the prior results of the MACS prevalence among the larger group of patients with apparently non-secretor adrenal tumours and cortisol following the administration of the standard 1 mg DST of <5 µg/dL [14].

In this study, we found that the total serum calcium was lower in the osteoporosis versus osteopenia group, respectively, versus the group with normal DXA (9.26 ± 0.52 versus 9.61 ± 0.41 mg/dL, *p* = 0.005, respectively, 9.79 ± 0.47 mg/dL, *p* < 0.001). However, the subjects showed similar parathormone and 25-hydroxyvitamin D levels. The mean values highlighted a mild hypovitaminosis D, which implies the menopausal status and subjects’ age rather than the presence of NFATs [30].

Notably, we did not rule out individuals undergoing vitamin D with/without calcium supplements, since this is a real-life setting in consecutive individuals with NFATs. Notably, the 25-hydroxyvitamin D assay provides the best insight of the overall metabolism of vitamin D [31].

Osteocalcin (31.95 ± 15.31 ng/mL) and CrossLaps (0.65 ± 0.27 ng/mL) were increased in patients with osteoporosis versus osteopenia [20.30 ± 8.71 ng/mL (*p* = 0.017), respectively of 0.41 ± 0.19 ng/mL (*p* < 0.001)] and to those with normal DXA [22.71 ± 10.56 ng/mL (*p* = 0.042), respectively of 0.47 ± 0.24 ng/mL (*p* = 0.036)]. This suggests a higher bone turnover in cases with lower BMD, which in group A showed additional interesting correlations between ACTH and CrossLaps, ACTH and 25-hydroxyvitamin D, ACTH and PTH, respectively, and between CrossLaps and cortisol after DST. Of note, type 2 diabetes might also influence the spectrum of the bone turnover markers profile, but its prevalence between the study groups was similar for this study [32].

The largest tumour diameter seemed important with respect to the bone status only in the group with a normal T-score at DXA by being associated with the level of osteocalcin (r = −0.427, *p* = 0.011), with P1NP (r = −0.427, *p* = 0.017) and with serum phosphorus (r = −0.467, *p* = 0.007). A larger diameter might be associated with a supressed bone turnover, which could be the precursor of an impaired BMD status at DXA (since this was the group with normal BMD).

Further on, the patients with confirmed osteoporosis are traditional candidates for anti-osteoporotic drugs according to general guidelines of menopausal osteoporosis [1]. Alternatively, a tailored strategy is required in cases with additional severe cardio-metabolic complications, tumour increase during surveillance or a tumour size larger than 4 cm. In these circumstances, a decision of adrenalectomy might be taken into consideration, with a potential post-operatory improvement in the bone status reducing the need for anti-osteoporotic drugs (yet not all studies agree) [33,34,35].

The limitations of this research are represented by the transversal design and the sample size. Further longitudinal prospective studies might help the understanding of the crossroads between bone status and NFATs in addition to taking into account a sample size calculation for an adequate (larger) analysis. The single-centre experience restricts the generalisability of the results; however, most similar studies on bone health amid NFATs are not very large cohorts. Moreover, we selected a homogenous population regarding the general fracture risk and did not include males or women of reproductive age who might show even more heterogeneous results of bone health status in terms of the bone mass at central DXA and circulating bone turnover biomarkers. Larger trials are necessary to analyse the confounders in the menopausal bone loss amid the co-presence of NFATs. Multifactorial algorithms are expected to estimate the fracture risk impact and become an important tool in this distinct endocrine landscape to pinpoint the patients who should benefit from immediate adrenalectomy versus those with long-term conservative surveillance in addition to anti-fracture medical and non-pharmacological strategies to address the osseous status, as well as the cardio-metabolic complications, which share biochemical and hormonal pathogenic loops (e.g., cortisol-ACTH status and glucose profile). Other types of elements to be combined with the bone–adrenal profile include the potential intervention and influence across lifestyle, e.g., physical exercise, nutrients and proteins intake, vitamin D supplementation, etc. On the other hand, additional information regarding imaging characteristics, other than the largest tumour diameter (Hounsfield units, lipid-rich adenomas, washout characteristics, etc.) would improve the endocrinological context of this study population.

## 5. Conclusions

This study emphasises a heterogeneous connection between the adrenal profile in NFATs and the clinical evaluation of menopausal bone status according to the various mineral metabolism assays and central DXA assessment. The rate of MACS and type 2 diabetes remains similar in the DXA categories, while traditional BMI’s influence on DXA-BMD represents the most important contributor in the presence of these tumours, irrespective of the adrenal hormonal and imaging parameters. More comprehensive prospective studies are mandatory to assess this multifactorial bone–adrenal interplay in order to improve the overall management.

## Figures and Tables

**Table 1:** Baseline demographic characteristics and adrenal and bone profile in the study population (N = 95).

Parameter	Entire Cohort (N = 95, 100%)	Group A (N = 23, 24.21%)	Group B (N = 45, 47.37%)	Group C (N = 27, 28.42%)	*p*-Value A–B	*p*-Value A–C	*p*-Value B–C	*p*-Value	Normal Range
Age (years), mean ± SD	61.59 ± 7.83	61.91 ± 6.86	62.98 ± 8.40	59.19 ± 7.42	0.603	0.186	0.058	0.140	
Years since menopause, mean ± SD	13.88 ± 8.24	15.86 ± 7.24	14.88 ± 8.61	10.94 ± 7.55	0.711	0.076	0.115	0.175	
Type 2 diabetes mellitus (N, %)	23 (24.21)	4 (17.39)	10 (22.22)	9 (33.33)	0.641	0.123	0.174	0.338	
High blood pressure (N, %)	74 (77.89)	15 (65.22)	37 (82.22)	22 (81.48)	0.118	0.168	0.993	0.267	
Dyslipidaemia (N, %)	75 (78.95)	15 (65.22)	38 (84.44)	22 (81.48)	0.070	0.168	0.796	0.174	
BMI (kg/sqm), mean ± SD	30.05 ± 6.01	26.90 ± 4.51	29.60 ± 4.89	33.24 ± 7.52	0.058	**0.003**	0.060	**0.004**	
**Adrenal profile**									
Bilateral (N, %)	28 (29.47)	8 (34.78)	10 (22.22)	10 (37.04)	0.264	0.754	0.120	0.374	
Largest tumour diameter (cm), mean ± SD	2.32 ± 0.98	2.29 ± 0.87	2.24 ± 1.06	2.48 ± 0.93	0.874	0.524	0.371	0.647	
ACTH (pg/mL), median (Q1, Q3)	11.88 (7.86, 16.28)	10.38 (8.38, 19.40)	12.65 (7.87, 16.15)	11.70 (6.93, 18.96)	0.569	0.741	0.327	0.598	3–66
Baseline morning plasma cortisol (µg/dL), mean ± SD	14.19 ± 6.53	12.46 ± 7.19	13.98 ± 5.30	16.08 ± 7.56	0.371	0.119	0.203	0.188	6.2–19.4
Plasma cortisol after 1 mg DST (µg/dL), median (Q1, Q3)	1.82 (1.13, 3.02)	1.56 (1.24, 2.69)	1.34 (1.02, 2.80)	2.20 (1.24, 4.79)	0.323	0.544	0.201	0.346	<1.8
MACS (N, %)	26 (27.37)	5 (21.74)	10 (22.22)	11 (40.74)	0.964	0.111	0.062	0.191	
**Biochemistry profile**									
Fasting glycaemia (mg/dL), mean ± SD	111.64 ± 44.80	120.11 ± 83.21	102.38 ± 12.98	118.32 ± 38.97	0.410	0.927	0.064	0.280	74–106
Glycated haemoglobin (%), mean ± SD	6.16 ± 1.22	6.02 ± 1.09	5.86 ± 0.55	6.72 ± 1.82	0.531	0.238	**0.050**	**0.049**	4.8–5.9
**Mineral metabolism and DXA evaluation**									
Total serum calcium (mg/dL), mean ± SD	9.58 ± 0.49	9.26 ± 0.52	9.61 ± 0.41	9.79 ± 0.47	**0.005**	**<0.001**	0.097	**<0.001**	8.4–10.2
Serum phosphorus (mg/dL), mean ± SD	3.70 ± 0.53	3.92 ± 0.63	3.62 ± 0.45	3.64 ± 0.55	**0.016**	0.112	0.881	**0.050**	2.5–4.5
Serum magnesium (mg/dL), mean ± SD	1.96 ± 0.22	1.90 ± 0.24	1.99 ± 0.22	1.96 ± 0.20	0.063	0.312	0.625	0.288	1.6–2.6
25-hydroxyvitamin D (ng/mL), mean ± SD	23.30 ± 10.29	23.74 ± 9.48	23.39 ± 10.45	23.06 ± 11.22	0.452	0.838	0.911	0.979	30–100
Parathormone (pg/mL), mean ± SD	44.65 ± 15.76	47.45 ± 16.87	42.88 ± 17.93	45.30 ± 11.91	0.231	0.679	0.613	0.699	15–65
Alkaline phosphatase (IU/L), mean ± SD	79.54 ± 30.32	85.53 ± 44.33	74.66 ± 19.87	83.32 ± 32.35	0.159	0.851	0.185	0.345	35–129
Osteocalcin (ng/mL), mean ± SD	23.54 ± 11.69	31.95 ± 15.31	20.30 ± 8.71	22.71 ± 10.56	**0.017**	**0.042**	0.375	**0.006**	14–46
P1NP (ng/mL), mean ± SD	54.94 ± 21.94	63.61 ± 11.95	56.52 ± 24.15	49.70 ± 22.35	0.440	0.111	0.373	0.303	20.25–76.31
CrossLaps (ng/mL), mean ± SD	0.48 ± 0.24	0.65 ± 0.27	0.41 ± 0.19	0.47 ± 0.23	**<0.001**	**0.036**	0.297	**0.004**	0.33–0.782
Lumbar BMD (g/cm^2^), mean ± SD	1.059 ± 0.168	0.861 ± 0.145	1.050 ± 0.115	1.196 ± 0.122	**<0.001**	**<0.001**	**<0.001**	**<0.001**	
Lumbar T-score (SD), mean ± SD	−1.12 ± 1.45	−2.68 ± 1.30	−1.06 ± 0.95	0.19 ± 0.92	**<0.001**	**<0.001**	**<0.001**	**<0.001**	>−1
Femoral neck BMD (g/cm^2^), mean ± SD	0.860 ± 0.150	0.656 ± 0.103	0.865 ± 0.090	1.000 ± 0.085	**<0.001**	**<0.001**	**<0.001**	**<0.001**	
Femoral neck T-score (SD), mean ± SD	−1.12 ± 0.13	−2.41 ± 0.69	−1.19 ± 0.56	−0.03 ± 0.68	**<0.001**	**<0.001**	**<0.001**	**<0.001**	>−1
Total hip BMD (g/cm^2^), mean ± SD	0.948 ± 0.175	0.760 ± 0.177	0.936 ± 0.141	1.083 ± 0.080	**0.002**	**<0.001**	**<0.001**	**<0.001**	
Total hip T-score (SD), mean ± SD	−0.61 ± 0.15	−2.07 ± 0.97	−0.56 ± 0.71	0.57 ± 0.72	**<0.001**	**<0.001**	**<0.001**	**<0.001**	>−1
Lowest T-score at central DXA (SD), mean ± SD	−1.56 ± 1.21	−3.17 ± 0.65	−1.54 ± 0.44	−0.23 ± 0.64	**<0.001**	**<0.001**	**<0.001**	**<0.001**	>−1
Trabecular bone score	1.318 ± 0.129	1.230 ± 0.138	1.291 ± 0.147	1.368 ± 0.094	0.535	0.063	0.159	0.177	>1.350
Prevalent fragility fracture (N, %)	6 (6.32)	3 (13.04)	2 (4.44)	1 (3.70)	0.199	0.481	0.622	0.412	
Vertebral	3 (50.00)	3 (100.00)	0	0					
Distal radius	1 (16.67)	0	1 (50.00)	0					
Femur	1 (16.67)	0	1 (50.00)	0					
Ankle	1 (16.67)	0	0	1 (100.00)					

**Table 2:** Age-group analysis among the patients with osteoporosis, osteopenia and normal DXA (N = 95).

Age Group (Years)	Total (N = 95, 100%)	Group A (N = 23, 24.21%)	Group B (N = 45, 47.37%)	Group C (N = 27, 28.42%)	*p*-Value
45–49	5 (5.26)	1 (4.35)	2 (4.44)	2 (7.41)	0.487
50–54	10 (10.52)	2 (8.70)	5 (11.11)	3 (11.11)	
55–59	23 (24.21)	5 (21.74)	7 (15.56)	11 (40.74)	
60–64	29 (30.53)	7 (30.43)	15 (33.33)	7 (25.93)	
65–69	16 (16.84)	6 (26.09)	7 (15.56)	3 (11.11)	
70–74	3 (3.16)	1 (4.35)	2 (4.44)	0 (0.00)	
75–79	9 (9.47)	1 (4.35)	7 (15.56)	1 (3.70)	

**Table 3:** Correlations between adrenal and bone profile in menopausal women diagnosed with osteoporosis at central DXA (N = 23).

Parameter	Osteocalcin (ng/mL)	Alkaline Phosphatase (U/L)	P1NP (ng/mL)	CrossLaps (ng/mL)	Lumbar BMD (g/cm^2^)	Femoral Neck BMD (g/cm^2^)	Total Hip BMD (g/cm^2^)	Lumbar T-Score (SD)	Femoral Neck T-Score (SD)	Total Hip T-Score (SD)	Total Serum Calcium (mg/dL)	Serum Phosphorus (mg/dL)	25-Hydroxyvitamin D (ng/mL)	Parathormone (pg/mL)
Baseline ACTH (pg/mL)	r = −0.128 *p* = 0.542	r = −0.226 *p* = 0.224	r = −0.214 *p* = 0.458	**r = −0.398** ***p* = 0.048**	r = −0.200 *p* = 0.392	r = −0.055 *p* = 0.815	r = −0.289 *p* = 0.245	r = −0.269 *p* = 0.245	r = 0.156 *p* = 0.523	r = −0.167 *p* = 0.396	r = −0.145 *p* = 0.404	r = −0.112 *p* = 0.519	**r = 0.448** ***p* = 0.020**	**r = −0.564** ***p* = 0.016**
Morning plasma (baseline) cortisol (µg/dL)	r = 0.154 *p* = 0.464	r = −0.037 *p* = 0.837	r = −0.214 *p* = 0.458	r = 0.022 *p* = 0.913	r = −0.182 *p* = 0.411	r = −0.333 *p* = 0.131	r = −0.418 *p* = 0.073	r = −0.272 *p* = 0.119	r = −0.033 *p* = 0.887	r = −0.147 *p* = 0.439	r = −0.035 *p* = 0.833	r = −0.018 *p* = 0.916	r = 0.317 *p* = 0.087	r = 0.055 *p* = 0.815
Second-day plasma cortisol after 1 mg DST (µg/dL)	r = −0.022 *p* = 0.929	r = −0.076 *p* = 0.731	r = −0.200 *p* = 0.573	**r = −0.550** ***p* = 0.019**	r = 0.429 *p* = 0.176	r = −0.048 *p* = 0.881	r = −0.071 *p* = 0.805	r = −0.140 *p* = 0.534	r = 0.001 *p* = 0.999	r = −0.142 *p* = 0.532	r = 0.271 *p* = 0.199	r = −0.013 *p* = 0.951	r = 0.200 *p* = 0.392	r = −0.422 *p* = 0.089
Largest tumour diameter (cm)	r = 0.322 *p* = 0.204	r = −0.258 *p* = 0.205	r = −0.048 *p* = 0.881	r = −0.019 *p* = 0.937	r = 0.225 *p* = 0.369	r = 0.056 *p* = 0.835	r = 0.225 *p* = 0.369	r = 0.335 *p* = 0.089	r = 0.464 *p* = 0.098	r = 0.219 *p* = 0.270	r = 0.205 *p* = 0.277	r = 0.179 *p* = 0.342	r = 0.039 *p* = 0.854	r = −0.092 *p* = 0.717

**Table 4:** Correlations between adrenal and bone profile in menopausal women diagnosed with osteopenia at central DXA (N = 45).

Parameter	Osteocalcin (ng/mL)	Alkaline Phosphatase (U/L)	P1NP (ng/mL)	CrossLaps (ng/mL)	Lumbar BMD (g/cm^2^)	Femoral Neck BMD (g/cm^2^)	Total Hip BMD (g/cm^2^)	Lumbar T-Score (SD)	Femoral Neck T-Score (SD)	Total Hip T-Score (SD)	Total Serum Calcium (mg/dL)	Serum Phosphorus (mg/dL)	25-Hydroxyvitamin D (ng/mL)	Parathormone (pg/mL)
Baseline ACTH (pg/mL)	r = −0.145 *p* = 0.288	r = −0.099 *p* = 0.420	**r = −0.367** ***p* = 0.048**	r = −0.210 *p* = 0.128	r = 0.030 *p* = 0.817	r = −0.034 *p* = 0.808	r = −0.121 *p* = 0.455	r = 0.006 *p* = 0.956	r = 0.022 *p* = 0.877	r = −0.067 *p* = 0.653	r = 0.147 *p* = 0.214	**r = −0.311** ***p* = 0.012**	r = −0.222 *p* = 0.091	r = 0.257 *p* = 0.103
Morning plasma (baseline) cortisol (µg/dL)	r = −0.094 *p* = 0.491	r = −0.093 *p* = 0.434	r = −0.083 *p* = 0.653	r = −0.124 *p* = 0.369	r = 0.091 *p* = 0.465	r = −0.103 *p* = 0.441	r = −0.221 *p* = 0.173	r = −0.022 *p* = 0.850	r = −0.112 *p* = 0.415	r = 0.026 *p* = 0.861	r = 0.210 *p* = 0.069	r = 0.175 *p* = 0.146	r = −0.153 *p* = 0.245	r = −0.162 *p* = 0.305
Second-day plasma cortisol after 1 mg DST (µg/dL)	r = −0.124 *p* = 0.472	r = 0.029 *p* = 0.856	r = 0.182 *p* = 0.411	r = 0.120 *p* = 0.494	r = −0.069 *p* = 0.673	r = 0.000 *p* = 0.999	r = −0.100 *p* = 0.589	r = 0.104 *p* = 0.491	r = 0.017 *p* = 0.928	r = −0.054 *p* = 0.760	r = −0.088 *p* = 0.560	r = 0.001 *p* = 0.999	r = −0.021 *p* = 0.897	r = −0.219 *p* = 0.255
Largest tumour diameter (cm)	r = 0.000 *p* = 0.999	r = 0.072 *p* = 0.539	r = 0.059 *p* = 0.752	r = 0.124 *p* = 0.369	r = −0.083 *p* = 0.495	r = 0.044 *p* = 0.734	r = 0.113 *p* = 0.463	r = 0.049 *p* = 0.665	r = 0.077 *p* = 0.565	r = 0.060 *p* = 0.674	r = −0.219 *p* = 0.055	r = −0.038 *p* = 0.754	r = −0.032 *p* = 0.803	r = 0.222 *p* = 0.150

**Table 5:** Correlations between adrenal and bone profile in menopausal women with normal BMD at DXA (N = 27).

Parameter	Osteocalcin (ng/mL)	Alkaline Phosphatase (U/L)	P1NP (ng/mL)	CrossLaps (ng/mL)	Lumbar BMD (g/cm^2^)	Femoral Neck BMD (g/cm^2^)	Total Hip BMD (g/cm^2^)	Lumbar T-Score (SD)	Femoral Neck T-Score (SD)	Total Hip T-Score (SD)	Total Serum Calcium (mg/dL)	Serum Phosphorus (mg/dL)	25-Hydroxyvitamin D (ng/mL)	Parathormone (pg/mL)
Baseline ACTH (pg/mL)	r = 0.088 *p* = 0.600	r = −0.033 *p* = 0.833	r = 0.059 *p* = 0.742	r = −0.095 *p* = 0.559	r = −0.081 *p* = 0.608	r = −0.098 *p* = 0.570	r = −0.207 *p* = 0.248	r = −0.082 *p* = 0.607	r = −0.195 *p* = 0.269	r = −0.082 *p* = 0.650	r = −0.145 *p* = 0.340	r = 0.229 *p* = 0.172	r = 0.029 *p* = 0.856	r = 0.279 *p* = 0.118
Morning plasma (baseline) cortisol (µg/dL)	**r = −0.391** ***p* = 0.019**	r = −0.105 *p* = 0.506	r = −0.221 *p* = 0.217	r = −0.254 *p* = 0.119	r = −0.134 *p* = 0.397	r = −0.328 *p* = 0.058	r = −0.126 *p* = 0.483	r = −0.106 *p* = 0.505	**r = −0.485** ***p* = 0.006**	r = −0.179 *p* = 0.321	r = −0.101 *p* = 0.507	r = −0.135 *p* = 0.420	r = −0.263 *p* = 0.097	r = 0.170 *p* = 0.343
Second-day plasma cortisol after 1 mg DST (µg/dL)	r = −0.156 *p* = 0.441	r = −0.034 *p* = 0.868	r = −0.267 *p* = 0.215	r = 0.079 *p* = 0.700	r = 0.099 *p* = 0.617	r = 0.078 *p* = 0.729	r = 0.184 *p* = 0.389	r = 0.089 *p* = 0.652	r = 0.048 *p* = 0.834	r = 0.079 *p* = 0.712	r = −0.052 *p* = 0.785	**r = −0.528** ***p* = 0.010**	r = 0.103 *p* = 0.586	**r = −0.515** ***p* = 0.020**
Largest tumour diameter (cm)	**r = −0.427** ***p* = 0.011**	r = −0.067 *p* = 0.672	**r = −0.426** ***p* = 0.017**	r = −0.176 *p* = 0.294	r = −0.126 *p* = 0.413	r = −0.117 *p* = 0.528	r = 0.037 *p* = 0.836	r = −0.118 *p* = 0.446	r = −0.094 *p* = 0.619	r = −0.119 *p* = 0.509	r = −0.189 *p* = 0.213	**r = −0.467** ***p* = 0.007**	r = −0.110 *p* = 0.487	r = −0.283 *p* = 0.126

**Table 6:** Multiple regression model for lumbar BMD prediction (N = 95).

	Lumbar BMD		
	B ± SE	β	*p*-Value
Constant	1.067 ± 0.347		**0.007**
Age (years)	−0.013 ± 0.007	0.003	0.994
Years since menopause	−0.006 ± 0.007	−0.362	0.417
Body mass index (kg/sqm)	0.014 ± 0.006	0.490	**0.030**
Baseline ACTH (pg/mL)	−0.015 ± 0.005	−0.710	**0.007**
Second-day plasma cortisol after 1 mg DST (µg/dL)	−0.010 ± 0.037	−0.081	0.185
Largest tumour diameter (cm)	−0.060 ± 0.043	−0.418	0.795
	R^2^ = 0.471		

**Table 7:** Multiple regression for femoral neck BMD prediction (N = 95).

	Femoral Neck BMD		
	B ± SE	β	*p*-Value
Constant	1.322 ± 0.334		**0.002**
Age (years)	−0.011 ± 0.007	−0.842	0.121
Years since menopause	0.004 ± 0.008	0.275	0.604
Body mass index (kg/sqm)	0.011 ± 0.005	0.454	**0.062**
Baseline ACTH (pg/mL)	−0.009 ± 0.006	−0.333	0.179
Second-day plasma cortisol after 1 mg DST (µg/dL)	−0.031 ± 0.034	−0.285	0.380
Largest tumour diameter (cm)	0.014 ± 0.039	0.108	0.720
	R^2^ = 0.583		

**Table 8:** Multiple regression model to predict total hip BMD in the study population (N = 95).

	Total Hip BMD		
	B ± SE	β	*p*-Value
Constant	0.853 ± 0.402		**0.052**
Age (years)	−0.006 ± 0.007	−0.391	0.387
Years since menopause	−0.001 ± 0.008	−0.040	0.931
Body mass index (kg/sqm)	0.020 ± 0.007	0.587	**0.011**
Baseline ACTH (pg/mL)	−0.007 ± 0.006	−0.291	0.260
Second-day plasma cortisol after 1 mg DST (µg/dL)	−0.058 ± 0.044	−0.434	0.216
Largest tumour diameter (cm)	0.054 ± 0.056	0.311	0.349
	R^2^ = 0.491		

## Data Availability

All the data are in the article.

## References

[1] Kanis J.A., Cooper C., Rizzoli R., Reginster J.Y., Scientific Advisory Board of the European Society for Clinical and Economic Aspects of Osteoporosis and Osteoarthritis (ESCEO) and the Committees of Scientific Advisors and National Societies of the International Osteoporosis Foundation (IOF) (2019). Executive summary of the European guidance for the diagnosis and management of osteoporosis in postmenopausal women. Calcif. Tissue Int..

[2] Gregson C.L., Armstrong D.J., Avgerinou C., Bowden J., Cooper C., Douglas L., Edwards J., Gittoes N.J.L., Harvey N.C., Kanis J.A. (2025). National Osteoporosis Guideline Group (NOGG). The 2024 UK clinical guideline for the prevention and treatment of osteoporosis. Arch. Osteoporos..

[3] Owei L., Wachtel H. (2025). The Landmark Series: Evaluation and Management of Adrenal Incidentalomas. Ann. Surg. Oncol..

[4] Poiana C., Musat M., Carsote M., Chirita C. (2009). Premenstrual dysphoric disorder: Neuroendocrine interferences. Rev. Med. Chir. Soc. Med. Nat. Iasi.

[5] Dumitru N., Ghemigian A., Carsote M., Albu S.E., Terzea D., Valea A. (2016). Thyroid nodules after initial evaluation by primary health care practitioners: An ultrasound pictorial essay. Arch. Balk. Med. Union.

[6] Manea M.M., Dragos D., Ghenu M.I., Enache I.I., Stoican I.C., Ciulavu C., Vasiliu O., Sirbu C.A., Tuta S. (2025). The Neurocardiogenic Impact of Ischemic Stroke: Intricacies of Cardiac Enzymes and the Vegetative System. Rom. J. Mil. Med..

[7] Savoie P.H., Murez T., Rocher L., Neuville P., Escoffier A., Fléchon A., Branger N., Camparo P., Rouprêt M. (2024). French AFU Cancer Committee Guidelines—Update 2024–2026: Assessment of an adrenal incidentaloma and oncological management. Fr. J. Urol..

[8] Suntornlohanakul O., Mandal S., Saha P., Saygili E.S., Asia M., Arlt W., Elhassan Y.S., Prete A., Ronchi C.L. (2024). Presentation and management of patients with adrenal masses: A large tertiary centre experience. Eur. J. Endocrinol..

[9] Mitrica M., Vasiliu O., Plesa A., Sirbu O.M. (2025). Multinodular and vacuolating neuronal tumor. Rom. J. Mil. Med..

[10] Albulescu D.M., Ionovici N., Moldovan H.R., Demetrian A.D., Bălă V.S., Constantin C., Bumbea A.M., Pănuş C., Albu V.C. (2017). Muscle metastases from cervical carcinoma—Case report. Rom. J. Morphol. Embryol..

[11] Janiak K., Józwik-Plebanek K., Kamiński G. (2024). Recent guidelines for diagnostic and therapeutic management of accidentally detected adrenal tumours (incidentaloma) in adults. Endokrynol. Pol..

[12] Yoshida Y., Horiuchi K., Otsuki M., Okamoto T. (2024). Diagnosis and management of adrenal incidentaloma: Use of clinical judgment and evidence in dialog with the patient. Surg. Today.

[13] Rowe N.E., Kumar R., Schieda N., Siddiqi F., McGregor T., McAlpine K., Violette P., Bathini V., Eng M., Izard J. (2023). Diagnosis, Management, and Follow-Up of the Incidentally Discovered Adrenal Mass: CUA Guideline Endorsed by the AUA. J. Urol..

[14] Fassnacht M., Tsagarakis S., Terzolo M., Tabarin A., Sahdev A., Newell-Price J., Pelsma I., Marina L., Lorenz K., Bancos I. (2023). European Society of Endocrinology clinical practice guidelines on the management of adrenal incidentalomas, in collaboration with the European Network for the Study of Adrenal Tumors. Eur. J. Endocrinol..

[15] Braun L.T., Vogel F., Zopp S., Marchant Seiter T., Rubinstein G., Berr C.M., Künzel H., Beuschlein F., Reincke M. (2022). Whom Should We Screen for Cushing Syndrome? The Endocrine Society Practice Guideline Recommendations 2008 Revisited. J. Clin. Endocrinol. Metab..

[16] Prete A., Bancos I. (2024). Mild autonomous cortisol secretion: Pathophysiology, comorbidities and management approaches. Nat. Rev. Endocrinol..

[17] Fernandes-Rosa F.L., Boulkroun S., Fedlaoui B., Hureaux M., Travers-Allard S., Drossart T., Favier J., Zennaro M.C. (2023). New advances in endocrine hypertension: From genes to biomarkers. Kidney Int..

[18] Abdellatif A.B., Fernandes-Rosa F.L., Boulkroun S., Zennaro M.C. (2022). Vascular and hormonal interactions in the adrenal gland. Front. Endocrinol..

[19] Iorio M., Celi M., Caliumi C., Cerci S., Cotesta D., Baldini V., Serra V., Petramala L., Diacinti D., D’Erasmo E. (2004). Turnover of bone metabolism and prevalence of vertebral fractures in adrenal incidentalomas. Recent. Prog. Med..

[20] Chen A.X., Burt M.G. (2023). Cardio-metabolic pathophysiology in mild glucocorticoid excess: Potential implications for management of adrenal incidentaloma. Clin. Endocrinol..

[21] Rebelo J.F.D., Costa J.M., Junqueira F.D., Fonseca A.O., de Almeida A.B.A.B.S., Moraes A.B., Vieira Neto L. (2023). Adrenal incidentaloma: Do patients with apparently nonfunctioning mass or autonomous cortisol secretion have similar or different clinical and metabolic features?. Clin. Endocrinol..

[22] Nakao H., Yokomoto-Umakoshi M., Nakatani K., Umakoshi H., Ogata M., Fukumoto T., Kaneko H., Iwahashi N., Fujita M., Ogasawara T. (2023). Adrenal steroid metabolites and bone status in patients with adrenal incidentalomas and hypercortisolism. eBioMedicine.

[23] Dos Santos Marques A.C., Brito B., Gorett Brito Fontes J., Reis Alves Carneiro G., Dickson Rebelo J.F., Barbosa Moraes A., Vieira Neto L., Costa Padilha M. (2025). Cortisol quantification in human plasma and urine by liquid chromatography coupled to mass spectrometry: Validation, analysis and application in a reference population and patients with adrenal incidentalomas. Clin. Chim. Acta.

[24] Zavatta G., Di Dalmazi G. (2024). Mild Autonomous Cortisol Secretion (MACS)—Related Osteoporosis. Exp. Clin. Endocrinol. Diabetes.

[25] Pal R., Banerjee M., Prasad T.N., Walia R., Bhadada T., Singh J., Bhadada S.K. (2024). Fracture risk and bone health in adrenal adenomas with mild autonomous cortisol secretion/subclinical hypercortisolism: A systematic review, meta-analysis and meta-regression. J. Bone Miner. Res..

[26] Remde H., Kimpel O., Fassnacht M. (2022). Adrenal incidentaloma—Differential diagnosis and management. Dtsch. Med. Wochenschr..

[27] Paschou S.A., Dede A.D., Anagnostis P.G., Vryonidou A., Morganstein D., Goulis D.G. (2017). Type 2 Diabetes and Osteoporosis: A Guide to Optimal Management. J. Clin. Endocrinol. Metab..

[28] Liu M.S., Lou Y., Chen H., Wang Y.J., Zhang Z.W., Li P., Zhu D.L. (2022). Performance of DHEAS as a Screening Test for Autonomous Cortisol Secretion in Adrenal Incidentalomas: A Prospective Study. J. Clin. Endocrinol. Metab..

[29] Kebebew E. (2021). Adrenal Incidentaloma. N. Engl. J. Med..

[30] Lerchbaum E. (2014). Vitamin D and menopause—A narrative review. Maturitas.

[31] Kim M.S., Kim T.H. (2024). Anti-Aging Tests for Middle Aged Women. J. Menopausal Med..

[32] Valea A., Carsote M., Moldovan C., Georgescu C. (2018). Chronic autoimmune thyroiditis and obesity. Arch. Balk. Med. Union.

[33] Morelli V., Favero V., Frigerio S., Aresta C., Pugliese F., Salcuni A.S., Risio A., Eller-Vainicher C., Palmieri S., Cairoli E. (2025). Adrenalectomy Reduces the Risk of Vertebral Fractures in Patients with Mild Autonomous Cortisol Secretion. J. Clin. Endocrinol. Metab..

[34] Zekan D., King R.S., Hajiran A., Patel A., Deem S., Luchey A. (2022). Diagnostic dilemmas: A multi-institutional retrospective analysis of adrenal incidentaloma pathology based on radiographic size. BMC Urol..

[35] Jackson B.S. (2023). Adrenal Incidentaloma Controversial Size Recommendations. Urol. Res. Pract..

